# Detection of Paratuberculosis in Dairy Herds by Analyzing the Scent of Feces, Alveolar Gas, and Stable Air

**DOI:** 10.3390/molecules26102854

**Published:** 2021-05-11

**Authors:** Michael Weber, Peter Gierschner, Anne Klassen, Elisa Kasbohm, Jochen K. Schubert, Wolfram Miekisch, Petra Reinhold, Heike Köhler

**Affiliations:** 1 Institute of Molecular Pathogenesis at ‘Friedrich-Loeffler-Institut’ (Federal Research Institute for Animal Health), Naumburgerstr. 96a, 07743 Jena, Germany; michael.weber@fli.de (M.W.); annekuentzel@hotmail.de (A.K.); petra.reinhold@fli.de (P.R.); 2 Rostock Medical Breath Research Analytics and Technologies (RoMBAT), Department of Anesthesia and Intensive Care, Rostock University Medical Center, Schillingallee 35, 18057 Rostock, Germany; peter.gierschner@gmx.net (P.G.); jochen.schubert@uni-rostock.de (J.K.S.); wolfram.miekisch@uni-rostock.de (W.M.); 3 Albutec GmbH, Schillingallee 68, 18057 Rostock, Germany; 4 Thüringer Tierseuchenkasse, Rindergesundheitsdienst (Thuringian Animal Health Fund, Cattle Health Service), Victor-Goerttler-Straße 4, 07745 Jena, Germany; 5 Department of Mathematics and Computer Science, University of Greifswald, Walther-Rathenau-Straße 47, 17489 Greifswald, Germany; elisa.kasbohm@uni-greifswald.de; 6 National Reference Laboratory for Paratuberculosis, Naumburger Straße 96a, 07743 Jena, Germany

**Keywords:** classification models, dairy cows, exhaled breath, fecal headspace, *Mycobacterium avium* ssp. *paratuberculosis* (MAP), paratuberculosis, random forest, stable air, volatile organic compound (VOC)

## Abstract

Paratuberculosis is an important disease of ruminants caused by *Mycobacterium avium* ssp. *paratuberculosis* (MAP). Early detection is crucial for successful infection control, but available diagnostic tests are still dissatisfying. Methods allowing a rapid, economic, and reliable identification of animals or herds affected by MAP are urgently required. This explorative study evaluated the potential of volatile organic compounds (VOCs) to discriminate between cattle with and without MAP infections. Headspaces above fecal samples and alveolar fractions of exhaled breath of 77 cows from eight farms with defined MAP status were analyzed in addition to stable air samples. VOCs were identified by GC–MS and quantified against reference substances. To discriminate MAP-positive from MAP-negative samples, VOC feature selection and random forest classification were performed. Classification models, generated for each biological specimen, were evaluated using repeated cross-validation. The robustness of the results was tested by predicting samples of two different sampling days. For MAP classification, the different biological matrices emitted diagnostically relevant VOCs of a unique but partly overlapping pattern (fecal headspace: 19, alveolar gas: 11, stable air: 4–5). Chemically, relevant compounds belonged to hydrocarbons, ketones, alcohols, furans, and aldehydes. Comparing the different biological specimens, VOC analysis in fecal headspace proved to be most reproducible, discriminatory, and highly predictive.

## 1. Introduction

Paratuberculosis (paraTb) is one of the four economically most important infectious diseases of dairy cattle [1] in developed countries. Caused by *Mycobacterium avium* subsp. *paratuberculosis* (MAP), it is a chronic progressive granulomatous enteritis resulting in malnutrition, reduction in milk yield, weight loss, and eventually, death. Infection with MAP occurs in young animals and may remain clinically nonapparent for several years until clinical signs are observed [2]. Identification of affected herds is crucial for successful control of the disease; however, the existing diagnostic methods have limitations, either regarding their sensitivity (antibody detection) or due to high expenditure of time and labor (bacterial culture, molecular biological detection via PCR).

During recent years, the development of diagnostic tests that allow a rapid, economic, and reliable identification of animals or herds affected by infectious diseases on the spot, so-called pen-side tests, received growing attention. Chromatography-based lateral flow tests, for example, were developed for the detection of viruses [3,4,5] or antigen-specific antibodies [6,7] in serum samples. However, the application of these tests demands invasive blood sampling. Analysis of volatile organic compounds (VOCs) present in exhaled breath and headspace air of fecal samples was suggested as a novel, alternative, noninvasive approach to the diagnosis of infectious diseases, in particular *Mycobacteria* infections [8,9,10,11]. The relevant VOCs originate from different sources within the host-pathogen interaction, such as the bacterial metabolism and the inflammatory and immunological host response to the pathogen [12]. They belong to all classes of organic substances and appear in very low concentrations (ppbV to pptV). Due to their physicochemical properties, they transform into a gaseous state already at low temperatures [13]. Discrimination between infected and noninfected animals is not based on individual compounds, but on relative concentration changes of different informative VOCs, thus demanding multivariate data analysis [14].

The contribution of VOCs of variable origin to indoor air quality and their impact on human health has been studied extensively [15]. However, the diagnostic potential of VOC analysis in stable air, the equivalent to indoor air in animal husbandry, has not been assessed so far. This would be another option for pen-side diagnosis.

This proof-of-principle study performed in eight dairy herds of different farms was based on the hypothesis that it should be possible to discriminate between cattle with and without MAP infections by using VOCs as biomarkers of infection. Which biological specimen turns out as the most suitable one to detect MAP infection under farming conditions remained to be elucidated. Thus, (i) headspaces above fecal samples, (ii) the alveolar fractions of exhaled breath, and (iii) stable air samples were analyzed in parallel and were compared with respect to their diagnostically relevant VOC patterns.

## 2. Results

### 2.1. Visualization of VOC Datasets from Feces, Alveolar Gas, and Stable Air

**Figure 1:**
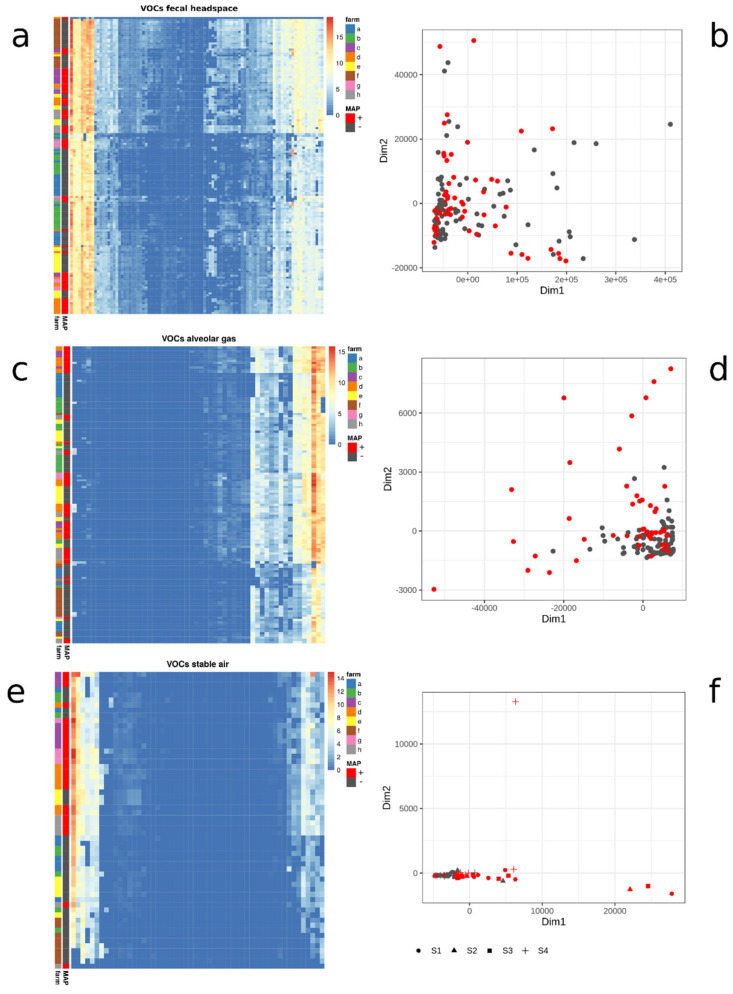
Heatmaps and two-dimensional scatterplots for each VOC dataset: fecal headspace (**a**,**b**), alveolar gas (**c**,**d**), and stable air (**e**,**f**). Heatmaps are composed of samples (rows), VOCs (columns), and two annotation columns. Heat colors indicate the log2-transformed substance concentration, which is explained by the colored range legend. Annotation columns indicate the associated farm and MAP status. Scatterplots display multidimensionally scaled (MDS) data points, each representing a VOC sample colored according to MAP status (red: MAP-positive, black: MAP-negative).

The first step of the data analysis involved the explorative visualization of the three multidimensional datasets: fecal headspace data (F), alveolar gas data (A), and stable air data (S). Each is represented by a matrix, composed of a number of measured VOCs (columns) and samples (rows), which are either cattle associated (A,F) or stable associated (S). Additionally, stable-associated samples were subgrouped into S1, S2, S3, and S4, according to the location of sampling within the stable (see Methods). We aimed to visualize these VOC concentration matrices in a compact and illustrative way by using annotated heatmaps, which are shown in Figure 1. Each heatmap displays the VOC concentration levels in combination with annotation columns, which provide information about the MAP status and the corresponding farm of the sample.

In comparison, the heatmap plots reveal differences in the distribution of VOC concentration levels in the three datasets. Alveolar gas and stable air data have a large proportion of columns with values at the lowest level of the plotted color range (blue), while the majority of VOCs appeared to be present in fecal data. In total, 76 VOCs were found at detectable levels in feces, compared to 30 in alveolar gas. In stable air, there were 24 VOCs detectable in S1 and S2 samples (both collected at head-level of the animals), compared to 25 VOCs for S3 (collected close to the floor) and 23 VOCs for S4 (collected distant from animals). The varying number of detectable VOCs for each dataset resulted in a distinct number of potential features for the subsequent classification approach. Additionally, the farm annotation column does not indicate a distinct clustering of samples according to their farm but rather an overall mixed grouping of samples.

We also examined the clustering of the samples in each dataset in a two-dimensional scatter plot by using multidimensional scaling (MDS). These plots enable the analysis of relative dissimilarities between the VOC samples and the inspection of the presence of cluster structures. Fecal headspace samples partially form a cluster (Figure 1b), while the remaining samples are distributed in multiple different directions. MAP-positive and MAP-negative samples cannot be assigned to separate locations in the plot. Similarly, most alveolar gas samples group within a large cluster (Figure 1d), however, the outer samples are mainly assigned to MAP-positive animals. In the stable air plot (Figure 1f), there is generally less variability compared to the former two plots. Here, we observed a subset of MAP-positive samples, which is scattered along the x-axis. In summary, the MDS plots revealed some trends and suggested that a simple unsupervised separation of MAP samples is not feasible. Therefore, we decided to apply a multivariate machine learning approach to generate a classification model that is based on the combination of multiple VOC profiles. We analyzed each dataset individually to evaluate its performance in the classification of MAP.

### 2.2. Identification and Reproducibility of Significant VOCs Present in Fecal Headspaces and Alveolar Gas

**Figure 2:**
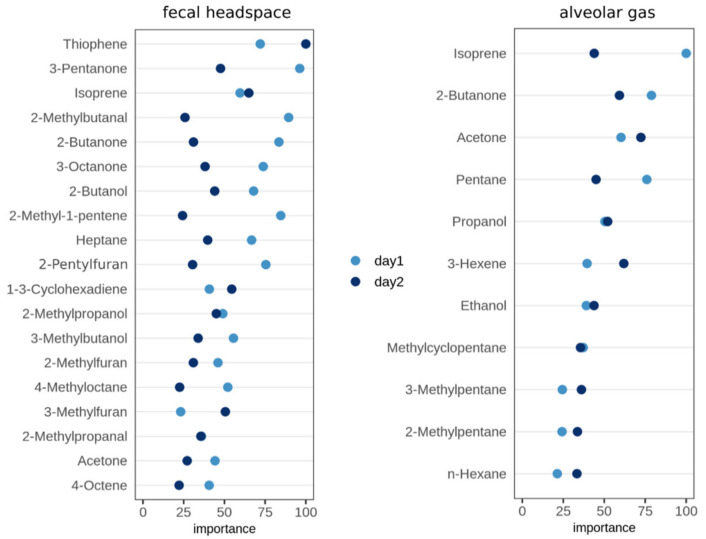
Variable importance plots displaying the importance values for all VOCs, which were selected with the Boruta method for each sampling day. The two panels show the resulting variables of the fecal headspace dataset and the alveolar gas dataset. The x-axis indicates the importance value, which is scaled to the maximum importance. Dot colors highlight the two sampling days.

Prior to the classification of the samples into MAP-positive and MAP-negative classes, we performed feature selection to identify VOCs that exhibit discriminatory and robust concentration levels. Therefore, we aimed to select VOCs that were measured across all farms and turned out as robust against the influence of the sampling time point. To investigate and quantify the latter temporal dependency, we performed the feature selection method Boruta on the two datasets representing headspace above fecal (F) and alveolar gas (A) samples. To compare the resulting importance scores, we generated dot charts, which are shown in Figure 2.

From the 76 detectable VOCs above fecal samples, 19 fulfilled the inclusion criteria, i.e., succeeded in the Boruta feature selection. In contrast, 11 out of 30 VOCs, were selected in the alveolar gas dataset. Generally, the selected subset of features from the first sampling day was confirmed by significant importance scores from the second sampling day. However, particularly in the fecal headspace dataset, some VOCs showed high variance in their importance rank, e.g., 2-methylbutanal and 2-methyl-1-pentene. This provided evidence that day-dependent deviations in the VOC levels exist, which needed to be taken into account by the classification approach. Therefore, the following classification model validation included separate model testing for both sampling days.

### 2.3. Classification of MAP Status from VOCs Present above Feces or in Alveolar Gas

**Figure 3:**
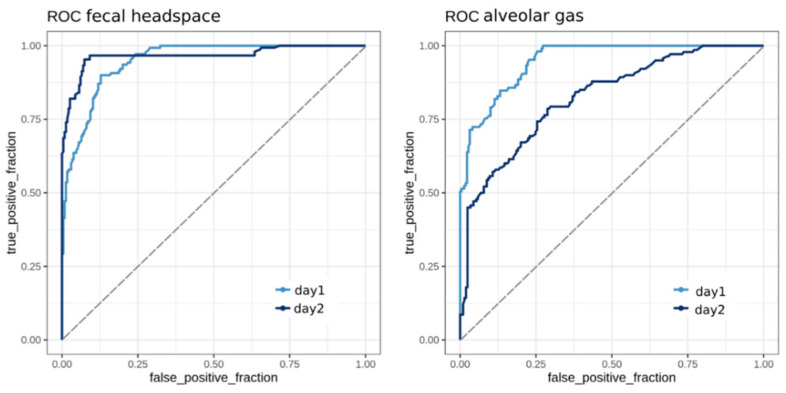
Receiver operating characteristic (ROC) curves for random forest classification models from subsets of fecal headspace data (model F, 19 variables) and alveolar gas data (model A, 11 variables). Each curve represents the outcome of repeated model cross-validation on unseen test data from either day 1 or day 2. Area under the curves (AUC) values for model F are 0.94 (day 1) and 0.96 (day 2) and for model A are 0.95 (day 1) and 0.82 (day 2).

Using the VOC concentration data from the subset of previously selected VOCs, we aimed to build one classification model for each dataset, which correctly predicts the MAP disease status of the samples. We employed random forest models to perform the classification as described in Methods. To estimate the accuracy of the resulting models, we generated receiver operating characteristic (ROC) curves, which are displayed in Figure 3.

Each curve represents the predictions of repeated 10-fold cross-validation for the respective dataset. To evaluate the model performance for both sampling days, we generated an individual ROC curve using the test predictions for each day. The area under the curves (AUC)–ROC values were calculated for all the curves.

The F-model (from fecal headspace samples) achieved comparably high levels (AUC–ROC day 1 = 0.94, AUC–ROC day 2 = 0.96), which indicated less model dependence on the sampling day and good reproducibility of the prediction accuracies. In contrast, the A-model (from samples of alveolar gas) showed promising results (AUC–ROC = 0.95) on day 1 but performed slightly worse on the second day (AUC–ROC = 0.82). Although the model was trained on data from both sampling days, it tended to predict day 1 samples more accurately, which in turn indicated that the VOC levels showed higher discriminatory power on the first day.

### 2.4. Identification of Significant VOCs Classifying for Paratuberculosis in Stable Air

In the next step, we analyzed the VOC composition in the stable air samples (collected at different locations) in order to investigate the variability and spatial dependencies of the VOC profiles. Hereby, we distinguished between four types of samples (S1, S2, S3, S4) as described in Methods. Initially, we analyzed if the presence of a face mask influenced the VOC profiles measured in front of the cow’s head. Therefore, we statistically compared the mean concentration between S1 and S2 for each VOC using Wilcoxon-Mann–Whitney tests. Since no significant differences were found (*p* > 0.05), we merged groups S1 and S2 and treated them as a single head-level group (S1S2).

**Figure 4:**
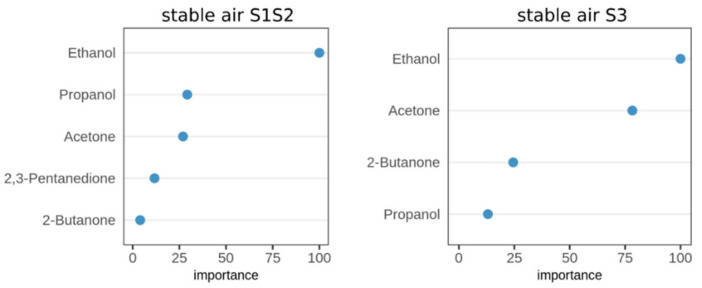
Variable importance plots displaying the importance values for all VOCs, which were selected with the Boruta method. The two panels show the resulting variables of the merged dataset of stable air collected at head level (S1S2), and the dataset of stable air collected close to the floor (S3). The x-axis indicates the importance value, which is scaled to the maximum importance.

Next, we conducted a feature importance analysis as in Section 2.1 for the three groups of stable air (S1S2, S3, S4). As a result, 5 out of 25 detectable VOCs were selected from the S1S2 dataset, compared to 4 out of 25 from the S3 group (close to the floor). The resulting VOCs are shown in Figure 4.

Interestingly, no feature was confirmed relevant in the S4 group (stable air sampled distant from animals). Thus, two stable air groups remained (S1S2, S3), which represented VOC levels at head level and close to the floor, respectively. Both datasets were used to train a predictive classification model in the next step.

### 2.5. Classification of MAP Status from VOCs Present in Stable Air

**Figure 5:**
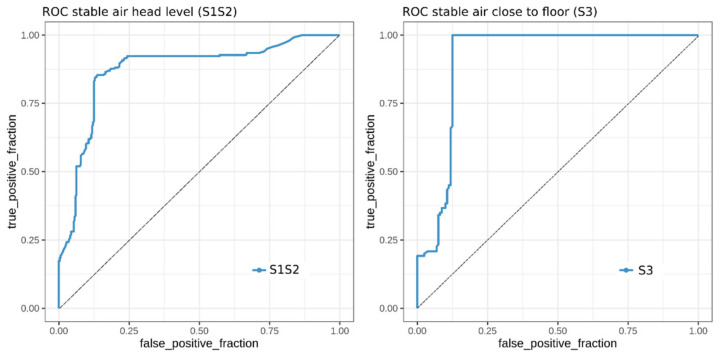
Receiver operating characteristic (ROC) curves for random forest classification models from subsets of stable air head-level data S1S2 (five variables) and stable air close to floor data S3 (four variables). Each curve represents the outcome of repeated model cross-validation. The area under the curve (AUC) for model S1S2 is 0.87 and for model S3 is 0.91.

In the second classification run, we aimed to build classification models from samples of two groups of stable air (S1S2, S3). Compared to the fecal headspace and alveolar gas datasets, the stable air dataset contains fewer samples (S1S2: n = 29, S3: n = 15); thus, we decided to perform cross-validation on all samples without creating separate datasets for each sampling day. Again, to evaluate the prediction performance of the model in cross-validation, we generated ROC curves for both models and calculated the associated AUC values. Stable air S1S2 achieved an averaged AUC value of 0.87, compared to an AUC value of 0.91 for S3 (Figure 5).

### 2.6. Comparison of Resulting VOC Sets

**Figure 6:**
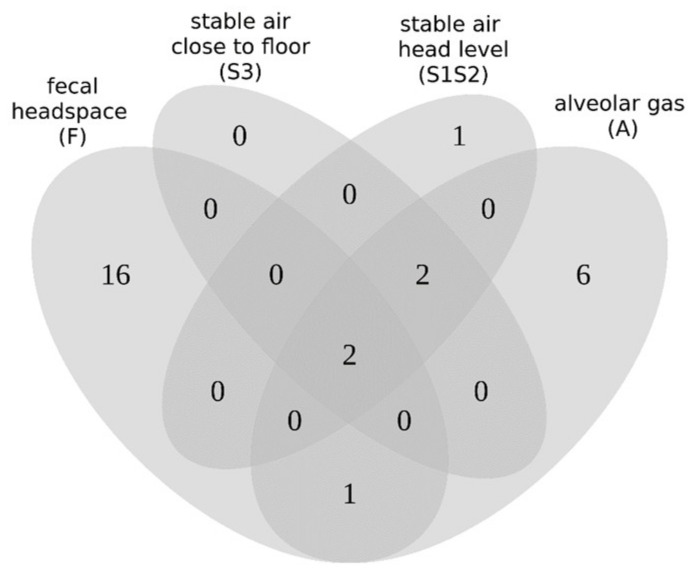
Venn diagram of four sets of selected VOCs (fecal headspace (F) n = 19, stable air close to floor (S3) n = 4, stable air head level (S1S2) n = 5, alveolar gas (A) n = 11), which represents the comparison of the selected VOCs. Values indicate the number of common VOCs among the sets of overlapping areas. The core area indicates the number of common VOCs, which are shared by all four sets (n = 2).

In total, we identified 28 VOCs that were found to be relevant for the classification of MAP samples across all investigated resources (Supplementary Materials, Table S1). Two compounds, i.e., acetone and 2-butanone, were detected under all four investigated conditions. Ethanol and propanol were detected in the groups of A, S1S2, and S3. Isoprene was present in fecal headspaces and alveolar gas samples. However, the largest number of VOCs was only present in a single set: 16 VOCs in fecal headspaces, 6 VOCs in alveolar gas, and 1 VOC in stable air collected close to the animals (S1S2). Since the Venn diagram (Figure 6) is based on the presence or absence of VOC compounds, we also evaluated the regulation of the VOCs and found that 26 VOCs were upregulated in MAP-positive samples, compared to MAP-negative samples under the tested conditions. Interestingly, 2-butanone was upregulated in groups A, S1S2, and S3 but downregulated in F. Additionally, 4-octene was downregulated in group F (Supplementary Materials, Table S1).

## 3. Discussion

Confirming the hypothesis that MAP infections in animals or herds, respectively, are detectable by means of volatile biomarkers, this study presents the first consideration of tracing VOC profiles as potential diagnostic markers even under field conditions of livestock farming. With respect to the suitability of different matrices, headspace above feces, the alveolar fractions of exhaled breath, and stable air samples revealed diagnostically relevant VOCs of unique but partly overlapping patterns. Biological and methodological aspects need to be taken into account when interpreting the results of this study.

### 3.1. Biological Aspects

MAP infection is characterized by a chronic local inflammation that mainly affects the gut-associated lymphoid tissue of the small intestine and proximal colon, the intestinal mucosa of jejunum and ileum, and the mesenteric and ileocolic lymph nodes. A wide variation of severity and distribution of lesions (focal to multifocal to diffuse), inflammatory cell infiltrates (lymphocytes, multinucleated giant cells, epitheloid macrophages), and mycobacteria within lesions (paucibacillary, multibacillary) of animals can be observed [16]. MAP is transferred into the intestinal content and is eventually shed within feces in varying concentrations [17]. Intestinal inflammation is accompanied by malabsorption and cachexia [18].

Based on these disease characteristics, we anticipated MAP-related VOCs predominantly in samples originating from feces acknowledging that (i) the pathogen itself and (ii) markers of intestinal inflammation and/or local immune response might contribute to the pattern of volatile compounds. Furthermore, the smell of feces will always be influenced by emissions related to the intestinal content, i.e., more or less digested feed components, cell debris of the mucosal epithelium, and constituents of the gut microbiota. Feed composition may vary between herds, but paraTb may also alter the intestinal interior due to inflammatory processes in the intestinal wall.

Exploiting the alveolar fraction of exhaled breath is based on the rationale that there is a huge contact surface between lung and blood allowing the transfer of blood-borne VOCs into the lung. Thus, alveolar gas is most likely representative of blood-borne volatiles. If all fractions of exhaled breath would be collected, a mixture between gas columns in the larger airways (airway dead space gas, composition roughly equivalent to ambient air) and alveolar gas would be analyzed. Compared to the human lung, the proportion of airway dead space volume per breath is much greater in large animals due to the anatomy of extra-thoracic airways [12]. Related to paraTb, blood-born VOCs may origin from (i) catabolic processes that are a consequence of malabsorption and inflammation and (ii) from systemic markers of inflammation and host response. In addition, processes localized in the gut or in draining lymph nodes as well as bacterial metabolism within the lesions might contribute to blood markers due to large blood–intestinal exchange areas within the body.

Both, VOC emissions from feces and from exhaled breath contribute to the VOC composition of stable air. However, the composition of stable air will be significantly dependent on the collection site within the stable.

#### 3.1.1. Sources of Variability in VOC Profiles

From the 76 detectable VOCs above fecal samples, 19 fulfilled the inclusion criteria, and 11 out of 30 VOCs were selected in the alveolar gas dataset. VOCs selected for the first sampling day were confirmed for the second day, although a certain degree of variation was observed. Based on these sets of VOCs, we were able to classify MAP-negative and MAP-positive samples with high accuracy. This confirms the findings of previous experimental studies suggesting that discrimination between MAP-negative and MAP-positive animals is possible by the analysis of the VOC profiles in the headspace of fecal samples and in exhaled breath, i.e., alveolar gas, of goats [8,14]. Despite standardized feeding, housing, and management conditions, physiological variability of discriminatory VOC profiles was noted [14]. The patterns of the most prominent substances changed in the course of infection; however, differences between inoculated and noninoculated animals remained detectable at any time in fecal samples and breath [8]. The results of two consecutive experimental studies in goats revealed that certain VOCs contributed reproducibly to the discriminatory VOC profile, although the effect size of the most important substances varied [14]. In contrast to standardized conditions in animal experiments, there are additional sources of variability of VOC emissions in the field, such as other diseases, differences in feed composition, bedding material, floor conditions, air exchange rates in the stable, numbers of animals per square meter. Such factors can influence the VOC measurements and therefore require the application of suitable statistical methods. Despite all these potential confounding factors, we successfully extracted VOC sets indicative of MAP infection from different sources.

#### 3.1.2. VOCs Indicative of MAP Infection

VOCs indicative of MAP infection differed between the three matrices explored in this study. Our results indicated that the number of VOCs contributing to discrimination was higher in fecal headspace (19 VOCs), compared to alveolar gas (11 VOCs), which is in line with previous reports from goat experiments [8,14]. The lowest number of discriminatory VOCs was observed in stable air (4–5 VOCs). Interestingly, some of the VOCs with high effect sizes in the goat experiments [14] were also considered important for the identification of MAP infection in cattle under field conditions, such as 2-butanone, acetone, and propanol in alveolar gas, and isoprene, 2-butanone, 3-octanone, heptane, 2-pentylfuran, 2-methylfuran, 3-methylfuran and acetone in fecal headspace. It seems that, in both animal species, these VOCs originate from similar metabolic and pathophysiologic processes.

There was only a partial overlap of indicative VOCs between the three matrices. Only two substances, acetone and 2-butanone, were generally considered important. Ethanol and propanol were discriminatory in alveolar gas and stable air, and isoprene in samples of alveolar gas and fecal headspace. As already discussed previously, VOCs indicative of MAP infection in fecal headspace and alveolar gas originate from different sources, which may explain these observations. Relevant VOCs in fecal headspace seemed predominantly related to MAP metabolism, in particular fatty acid turnover and carbon metabolism. This is supported by the fact that eight compounds, namely, heptane, 3-methylbutanol, acetone, 2-butanone, 3-pentanone, 3-octanone, 3-methylfuran, and 2-pentylfuran, belonged to the recently published VOC core profile of cultivated MAP strains [19]. Furthermore, five compounds, namely 2-methylpropanol, 3-methylbutanol, 3-pentanone, isoprene, and 2-methylfuran, were indicative for MAP growth during cultural isolation from clinical samples of cattle and goats [20].

In alveolar gas, however, compounds originating from inflammatory processes seemed to predominate. VOCs indicative of inflammatory bowel disease (IBD), such as 1-propanol, ethanol, and pentane [21], were also indicative of MAP infection. The local inflammatory processes in the intestine share features in both diseases. Inflammation may lead to oxidative stress and increased production of reactive oxygen species (ROS). ROS oxidize biologically important molecules and cause lipid peroxidation of polyunsaturated fatty acids, generating alkanes and methylated alkanes [22], such as pentane, hexane, 2- and 3-methylpentane, and methylcyclopentane, which belonged to the most important discriminatory substances in alveolar gas of MAP-infected cows in our study.

Some of the indicative VOCs, in particular acetone and isoprene, are likely to originate from both, mycobacterial metabolism and host response. Mycobacteria are able to produce methyl ketones, such as acetone [23], but acetone has also been linked to fat catabolism in cattle and humans [24,25]. One major source of isoprene is the bacterial metyl-erythritol phosphate pathway [26,27]; on the other hand, isoprene formation in humans was shown to correlate with cholesterol biosynthesis [28] and, more importantly, with IBD [29].

Stable air samples only turned out meaningful when the collection was performed close to the animals, i.e., either in front of the animal’s head (breathing area) or near to the stable’s floor (coated with feces or manure). Despite these different sites of collection, the interesting VOCs were nearly identical (Figure 4). However, compared to fecal headspaces or alveolar gas samples, the number of indicative VOCs found in relevant stable air samples was significantly lower. Multiple sources contribute to the VOC composition of stable air, such as emissions from feces, urine, and skin of the animals, gases eructated from the forestomaches of ruminants, components of breath, feedstuffs, bedding material, fuel, building materials, and VOCs contained in outdoor air. This may impair the identification of relevant VOCs. In addition, stable air is continuously exchanged by fresh outdoor air, which may result in a decrease of VOC concentrations below the limit of detection even in the vicinity of the animals.

### 3.2. Methodological Aspects

Sampling was conducted by preconcentration of VOCs either directly in the stable (alveolar gas and stable air) or from fecal headspace after filling feces into sealed headspace vials. Volatiles were identified later offline by GC–MS. This enabled the detection of VOCs in very low concentrations in the range of ppbV to pptV. Utilization of VOC analysis for practical pen-side diagnosis would demand a different approach. VOC emission has to be measured directly on the spot. Analytical platforms that allow an online analysis of VOC emissions, such as ion mobility spectrometry (IMS), ion flow tube–mass spectrometry (SIFT–MS), or proton transfer reaction–mass spectrometry (PTR–MS), respectively, are available and could be adapted for this purpose. Finally, the discriminatory performance of the adapted analysis systems compared to established diagnostic methods has to be evaluated.

Demands on test quality largely depend on the final purpose of testing, either identification of MAP-positive herds or individual MAP-positive animals. Analysis of fecal headspace or alveolar gas would enable identification of both, positive individuals as well as positive herds. Analysis of stable air would only allow identification of positive herds.

From the diagnostic perspective, test accuracy and validity of the classification model are of particular importance. Here, we applied an adapted multivariate classification analysis based on the random forest method, which has been used in various cases in the field of metabolomics [20,30,31]. In this context, some considerations were taken into account. First, overfitting of the predictive models, i.e., the loss of general prediction validity can occur if the model fitted too tightly to the training data. Random forests employ several techniques to avoid or minimize overfitting, for instance, the random selection of variables at each node, the error estimation on the unseen out-of-bag data, and the large number of trees, each of which is only based on a subset of the input data. Additionally, we applied repeated cross-validation to improve the error estimation of the model, due to the varying composition of the training dataset. This subsetting approach is essential to account for the variability of different animals, farms, and sampling days on the classification results. In this context, we also evaluated the reproducibility between consecutive measurement days. For validation, we generated model test sets for both days separately in order to evaluate one model on test data from two time points. Nevertheless, the validation of the model on an independently sampled population remains to be shown.

With AUC values of 0.94 (day 1) and 0.96 (day 2), VOC analysis in fecal headspace proved to be more reproducible and discriminatory than alveolar gas analysis for the identification of MAP-positive animals. Furthermore, analysis of fecal headspace was highly predictive, because the ROC curves closely approach the upper left corner of optimal prediction. VOC analysis in alveolar gas was less reproducible, because of marked differences in the AUC values between the two analysis days (0.95 on day 1 vs. 0.82 on day 2), and less predictive, too.

The AUC values calculated for VOC analysis in fecal headspace of individual cattle indicate that this approach has higher discriminatory power than established indirect antibody ELISAs for the identification of MAP-infected animals, where AUC values of 0.55–0.57 [32] or 0.77–0.91 [33] were calculated, depending on the reference method used. It is also superior to recently published novel biomarker-based diagnostic tests for paratuberculosis (ABCA13-based ELISA, SPARC ELISA, MMP8 ELISA) with AUC values of 0.79–0.85 [34]. However, these are only preliminary data, which have to be confirmed in future field studies covering larger numbers of animals and herds. Likewise, the number of MAP-positive and MAP-nonsuspect herds included in this study is too low to evaluate the diagnostic performance of the approach on the herd level.

This limitation also applies to VOC analysis in stable air. However, our preliminary data indicate that, despite relatively high AUC values (0.87 and 0.91), VOC analysis in stable air is not predictive enough for the identification of MAP-positive herds. This is mainly due to the fact that sensitivity is increased only to the cost of specificity, and a high rate of false-positive results has to be anticipated.

## 4. Animals, Materials, and Methods

### 4.1. Legislation and Ethical Approval

This study was carried out in strict accordance with European and National Law for the Care and Use of Animals. The protocol was approved by the Animal Health and Welfare Unit of the Thüringer Landesamt für Verbraucherschutz (Permit Number: 04-102/16; date of permission: 20.04.2016). The experiments were conducted under the supervision of the authorized institutional Animal Protection Officer. Every effort was made to minimize discomfort and suffering throughout the duration of the study. No sedation or anesthesia was applied to the animals.

### 4.2. Characteristics of Herds and Animals Used for Sample Collection

In total, 8 dairy herds and 77 individual dairy cows were included in this explorative study. All herds were enrolled in the voluntary paratuberculosis control program of the German federal state of Thuringia. The paratuberculosis status of the herds was defined depending on the results of annual whole herd tests for the presence of MAP in individual fecal samples by bacterial culture (fecal culture). Accordingly, four herds were classified as MAP-positive herds, because MAP was detected in the fecal samples of animals from these herds, and four herds were classified as MAP-nonsuspect herds, because MAP was not identified in any fecal sample of animals from these herds during at least three preceding annual whole herd tests.

The individual dairy cows included in this study were selected based on their fecal culture results during whole herd testing. Animals from MAP-nonsuspect herds were classified as MAP negative. Cows from MAP-positive herds with at least one positive test result of fecal culture were classified as MAP positive. During the study period, 30 MAP-positive cows were available, and 47 MAP-nonsuspect cows could be recruited. The animals were examined for fecal shedding of MAP at the time of VOC sampling by fecal culture [35]; and for the presence of antibodies against MAP in blood serum by a commercially available enzyme-linked immunosorbent assay (IDEXX Paratuberculosis Screening ELISA, IDEXX, Montpellier, France).

### 4.3. Sampling and Preconcentration of Headspace above Feces, Alveolar Gas, and Stable Air 

Each animal and each herd were sampled twice in a one-week interval. The numbers of samples included in this study are given in Table 1 and Table 2.

Fecal samples (F) were collected on an individual basis directly in a clean sampling container, either through rectal manipulation or during spontaneous defecation. From this, smaller portions of about 3 g of fresh feces per cow and time point were filled into a 20 mL headspace vial sealed with Teflon-coated rubber septa in combination with magnetic crimp caps, as described elsewhere [36]. The vials were stored at 4 °C and processed within 24–36 h after sampling. For preconcentration with needle trap microextraction (NTME), the vials were heated up to 37 °C. A needle trap device (NTD, packed with 1 cm divinylbenzene, Carbopack X and Carboxen) was connected to a 1 mL syringe and inserted through the Teflon-coated rubber. One mL of headspace gas was manually moved through the NTD into the syringe and back through the NTD into the headspace volume 20 times.

Collecting alveolar gas (A) was based on CO_2_-controlled sampling of exhaled breath using a tightly fitting face mask and combining mainstream capnometry with needle-trap microextraction (NTME). The technical setup designed particularly for large animals has been described in detail elsewhere [12,36,37]. Shortly, a fast responding capnometer ensured continuous measurement of CO_2_ in exhaled breath. Above a defined threshold (about 25–30 mm Hg or 3.3–4.0 kPa, respectively) a CO_2_-triggered valve opened and predominantly alveolar gas was directed through an NTD for collection of VOCs.

Stable air (S) was also collected using the NTME sampling device while 60 mL of stable air was enriched per sample without CO_2_-controlled opening of the valve. Stable air samples resulted from different locations:

- S1: at the level of the cow’s head, without the presence of any facemask;
- S2: in front of the cow’s head while wearing the facemask (used for exhaled breath sampling);
- S3: in the area close to the floor of the stable, i.e., close to emissions from feces, manure, or slurry;
- S4: in the area above the animals, far away from slurry or wastes, and partially floated naturally by fresh air.

### 4.4. Analysis and Biochemical Identification of VOCs

VOC analyses were performed using gas chromatography–mass spectrometry (GC–MS) at least 60 h after sampling. After thermodesorption of the NTDs, VOCs were separated with a GC (Agilent 7890A, Agilent Technologies, Boeblingen, Germany) using helium as carrier gas and detected by a mass spectrometer (Agilent 5975C inert XL MSD). The NIST Database (NIST 2005 Gatesburg, PA, USA) was used to identify the VOCs via the resulting mass spectra. Pure reference substances were measured to verify the identified VOCs by retention time and respective mass spectra. Quantification of all these VOCs was performed using a liquid calibration unit (LCU, Ionicon Analytik GmbH, Innsbruck, Austria). Humid standard gas mixtures were created in different concentrations from pure reference substances for calibration. Limit of detection and quantification was calculated by measuring the baseline of 10 blank samples. Concentrations below LOQ (limit of quantification) were set to zero.

### 4.5. Data Preprocessing

All resulting data files were loaded as tables into the R statistical environment (v3.6.1) [38] for further processing and analyses. For each dataset (feces, alveolar gas, stable air), a matrix of concentration values, along with annotation data, was processed. Annotation of each sample included identifiers for animal, farm, and day of measurement. We compared the distribution of the VOC measurements across the different samples to examine the presence of outliers. To visualize the multivariate concentration data (rows: samples, columns: VOCs), we generated hierarchically clustered heatmaps using the R package pheatmap (v1.0.8) [39]. This package allows for combined plotting of measurements and annotation columns, which indicate farm and MAP status in our plots. To account for the large numerical range of the data, we applied a log2 transformation (of x + 1) to obtain values in a suitable color scale. Furthermore, multidimensional scaling (MDS) was conducted with the help of the cmdscale function in R. This method returns a two-dimensional approximation of the pairwise sample distances. These transformed data points can be visualized in a scatter plot to examine the cluster structure of the data.

### 4.6. Feature Selection

Feature selection was performed to select a subset of suitable VOCs for the construction of a MAP classification model. Therefore, all measured compounds were tested using the method Boruta (v 6.0) [40]. This algorithm implements a permutation scheme to rank the variables with regard to their importance. Feature selection was performed for each dataset (fecal headspace, alveolar gas, stable air) and each measurement day separately. Importance values were scaled to the maximum value in order to obtain percentages. Features with confirmed importance of >25% were selected. Those features were used in the following classification approach.

In the analysis of alveolar gas data, butanoic-acid-Pr.E and propanoic-acid-2OH-EE were selected. Due to the well-known fact that short-chain fatty acids (i.e., mainly acetate, lactate, propionate, and butyrate) are the main products of fermentation processes in the forestomach system of ruminants, and that forestomach gases may alter exhaled breath composition significantly [12,41], these two VOCs were considered as “confounding compounds,” and were excluded from further statistical analyses.

### 4.7. Classification Model Building

To discriminate MAP-positive from MAP-negative samples, we used the supervised-learning approach random forest [42]. Here, we applied the R package randomForest (v4.6_14) in combination with the package caret (v6.0_83), which provides standardized procedures to perform training and cross-validation [43]. The combination of decision trees in a random forest is a well-established and robust approach to the classification of multivariate samples. Each tree is trained on a different resampled subset of the data and a different subset of variables. This random and highly independent learning phase is followed by the prediction of unseen samples (out-of-bag samples), which is implemented by a class vote of all trees. For each sample, the resulting predicted class is typically the one with the highest number of votes.

The caret package provides helpful functions to perform cross-validation and parameter optimization of the randomForest model [43]. Here, we applied repeated cross-validation (10-fold, 5 repeats) to obtain an averaged accuracy estimation of the final model. The parameter ntree (number of trees) was set to its default value (ntree = 500), while mtry (number of candidate variables in each split node) was separately optimized in each classification run. To evaluate the robustness of the resulting model, we modified the caret cross-validation procedure to perform model testing on data from both sampling days separately. Due to the limited number of samples, all available data were used in the cross-validation procedure without retaining samples for an external validation set.

The performance of the model predictions was evaluated in a receiver operating characteristic (ROC) analysis. Areas under the ROC curve (AUC–ROC) values were computed to compare the relationship between true positive rate and false positive rate (pROC package). Receiver operating characteristic (ROC) curves are used most commonly as a means of evaluating diagnostic tests. These curves are generated by plotting the sensitivity (true-positive rate) on the y-axis and 1-specificity (false-positive rate) on the x-axis. Curves that approach closest to the coordinate (x = 0, y = 1) are more highly predictive, whereas ROC curves that lie close to the line of equality indicate that the result is not better than obtained by chance. The area under the curve (AUC) is used to quantify the overall ability of a test to discriminate between two outcomes [44].

## 5. Conclusions

This explorative study revealed, for the first time, that VOC profiles can be used as potential diagnostic markers even under field conditions of livestock farming. Sets of VOCs indicative of MAP infection in dairy herds were successfully extracted from different biological matrices, i.e., above feces, in alveolar gas, and even in stable air. Discrimination between MAP-infected and -noninfected cattle was based on multivariate analysis of different VOCs with sufficient variable importance. The number of relevant VOCs was higher in fecal headspace (19 VOCs), compared to alveolar gas (11 VOCs) or stable air (4–5 VOCs). Due to different sources of origin, the relevant VOCs vary between different biological matrices, and further research is needed to correlate features of the disease (for example status of the infection, bacterial load, degree of local inflammation, systemic pathophysiological consequences, host response, etc.) with corresponding VOC pattern.

Among these three biological matrices, the headspace above feces turned out as most reproducible, discriminatory, and highly predictive. The diagnostic performance of fecal headspace analysis seemed to be similar or even superior to established and novel diagnostic tests for MAP infection. Despite these encouraging results, more field studies are necessary (covering larger numbers of animals and herds, and other animal species susceptible for MAP) to confirm the suitability of VOC analysis as a potential diagnostic test. Introducing VOC analysis as a practical pen-side diagnostic test would ideally require online analysis of VOC emissions. The performance of such analytic systems, compared to established diagnostic methods, has yet to be evaluated.

## Figures and Tables

**Table 1:** Numbers of alveolar gas and fecal samples obtained from dairy herds and included in VOC analyses.

Biological Specimen	Herd Status: MAP-Negative Herds/Animals/Samples	Herd Status: MAP-Positive Herds/Animals/Samples
alveolar gas	4/46/85	4/30/49
headspace above feces	4/47/93	4/30/58

**Table 2:** Numbers of stable air samples obtained from dairy herds and included in VOC analyses.

Locations of Stable Air Sampling	Herd Status: MAP-Negative Herds/Samples	Herd Status: MAP-Positive Herds/Samples
S1: head level, without face mask	4/8	4/7
S2: head level, through face mask	4/8	4/6
S3: close to floor contaminated with manure or feces	4/8	4/8
S4: distant from animals, slurry, or wastes, partially floated by fresh air	4/8	4/7

## Data Availability

Data are contained within the article or supplementary material.

## Supplementary Materials

The following are available online, Table S1: List of important VOCs.
