# Supplementary material for: Detection of Paratuberculosis in Dairy Herds by Analyzing the Scent of Feces, Alveolar Gas, and Stable Air
Source: Molecules. 2021 May 11;26(10):2854. doi: 10.3390/molecules26102854 (PMC8150929; doi:10.3390/molecules26102854)
Supplement: Supplementary file 1 [file molecules-26-02854-s001.zip › molecules-1201314-supplementary.pdf]

Supplement

Table S1. List of important VOCs.

number	VOC	chemical class	presence in biological samples	quantitative changes related to MAP-infection			
				fecal headspace	alveolar gas	stable air (S1S2)	stable air (S3)
1	2-methylpentane	hydrocarbon	alveolar gas		+		
2	3-hexene	hydrocarbon	alveolar gas		+		
3	3-methylpentane	hydrocarbon	alveolar gas		+		
4	methylcyclopentane	hydrocarbon	alveolar gas		+		
5	n-hexane	hydrocarbon	alveolar gas		+		
6	pentane	hydrocarbon	alveolar gas		+		
7	1-3-cyclohexadiene	hydrocarbon	fecal headspace	+			
8	2-butanol	alcohol	fecal headspace	+			
9	2-methyl-1-pentene	hydrocarbon	fecal headspace	+			
10	2-methylbutanal	aldehyde	fecal headspace	+			
11	2-methylfuran	furan	fecal headspace	+			
12	2-methylpropanal	aldehyde	fecal headspace	+			
13	2-methylpropanol	alcohol	fecal headspace	+			
14	2-penthylfuran	furan	fecal headspace	+			
15	3-methylbutanol	alcohol	fecal headspace	+			
16	3-methylfuran	furan	fecal headspace	+			
17	3-octanone	ketone	fecal headspace	+			
18	3-pentanone	ketone	fecal headspace	+			
19	4-methyloctane	hydrocarbon	fecal headspace	+			
20	4-octene	hydrocarbon	fecal headspace	-			
21	heptane	hydrocarbon	fecal headspace	+			
22	thiophene	hydrocarbon	fecal headspace	+			
23	isoprene	hydrocarbon	alveolar gas, fecal headspace	+	+		
24	2,3-pentanedione	ketone	stable air (S1S2)			+	
25	ethanol	alcohol	alveolar gas, stable air (S1S2, S3)		+	+	+
26	propanol	alcohol	alveolar gas, stable air (S1S2, S3)		+	+	+
27	2-butanone	ketone	alveolar gas, fecal headspace, stable air (S1S2, S3)	-	+	+	+
28	acetone	ketone	alveolar gas, fecal headspace, stable air (S1S2, S3)	+	+	+	+

Legend

+	mean MAP-positive > mean MAP-negative
-	mean MAP-positive < mean MAP-negative
